# Teleological role of L-2-hydroxyglutarate dehydrogenase in the kidney

**DOI:** 10.1242/dmm.045898

**Published:** 2020-11-27

**Authors:** Garrett Brinkley, Hyeyoung Nam, Eunhee Shim, Richard Kirkman, Anirban Kundu, Suman Karki, Yasaman Heidarian, Jason M. Tennessen, Juan Liu, Jason W. Locasale, Tao Guo, Shi Wei, Jennifer Gordetsky, Teresa L. Johnson-Pais, Devin Absher, Dinesh Rakheja, Anil K. Challa, Sunil Sudarshan

**Affiliations:** 1 Department of Urology, University of Alabama at Birmingham, Birmingham, AL 35294, USA; 2 Department of Biology, Indiana University, Bloomington, IN 47405, USA; 3 Department of Pharmacology and Cancer Biology, Duke University, Durham, NC 27710, USA; 4 Department of Pathology, University of Alabama at Birmingham, Birmingham, AL 35294, USA; 5 Departments of Pathology and Urology, Vanderbilt University Medical Center, Nashville, TN 37232, USA; 6 Department of Urology, UT Health San Antonio, San Antonio, TX 78229, USA; 7 HudsonAlpha Institute for Biotechnology, Huntsville, AL 35806, USA; 8 Department of Pathology, University of Texas Southwestern Medical Center, Dallas, TX 75390, USA; 9 Department of Biology, University of Alabama at Birmingham, Birmingham, AL 35294, USA; 10 Birmingham VA Medical Center, Birmingham, AL 35233, USA

**Keywords:** L-2-hydroxyglutarate, L-2-hydroxyglutarate dehydrogenase, TCA cycle, PPARGC1A

## Abstract

L-2-hydroxyglutarate (L-2HG) is an oncometabolite found elevated in renal tumors. However, this molecule might have physiological roles that extend beyond its association with cancer, as L-2HG levels are elevated in response to hypoxia and during *Drosophila* larval development. L-2HG is known to be metabolized by L-2HG dehydrogenase (L2HGDH), and loss of L2HGDH leads to elevated L-2HG levels. Despite L2HGDH being highly expressed in the kidney, its role in renal metabolism has not been explored. Here, we report our findings utilizing a novel CRISPR/Cas9 murine knockout model, with a specific focus on the role of L2HGDH in the kidney. Histologically, *L2hgdh* knockout kidneys have no demonstrable histologic abnormalities. However, GC-MS metabolomics demonstrates significantly reduced levels of the TCA cycle intermediate succinate in multiple tissues. Isotope labeling studies with [U-^13^C] glucose demonstrate that restoration of L2HGDH in renal cancer cells (which lowers L-2HG) leads to enhanced incorporation of label into TCA cycle intermediates. Subsequent biochemical studies demonstrate that L-2HG can inhibit the TCA cycle enzyme α-ketoglutarate dehydrogenase. Bioinformatic analysis of mRNA expression data from renal tumors demonstrates that L2HGDH is co-expressed with genes encoding TCA cycle enzymes as well as the gene encoding the transcription factor PGC-1α, which is known to regulate mitochondrial metabolism. Restoration of PGC-1α in renal tumor cells results in increased L2HGDH expression with a concomitant reduction in L-2HG levels. Collectively, our analyses provide new insight into the physiological role of L2HGDH as well as mechanisms that promote L-2HG accumulation in disease states.

## Abstract (precis)

**Summary:** Using a murine knockout model and analysis of human cells, we demonstrate a novel role for L-2-hydroxyglutarate dehydrogenase in the kidney as well as new insight into the mechanisms that regulate expression of this enzyme.

## INTRODUCTION

Oncometabolites are small molecules that have been found elevated in various malignancies. To date, these molecules include the tricarboxylic acid (TCA) cycle intermediates succinate and fumarate as well as both enantiomers of 2-hydroxyglutarate (D-2HG and L-2HG) (Tomlinson et al., 2002; Baysal et al., 2000; Niemann and Müller, 2000; Parsons et al., 2008; Yan et al., 2009; Green and Beer, 2010; Mardis et al., 2009). Notably, elevated oncometabolite levels are observed with inborn errors of metabolism, such as elevated fumarate caused by fumarase deficiency. Both forms of 2HG may be elevated in the setting of acidurias related to loss-of-function mutations in the D-2HG dehydrogenase (*D2HGDH*) or L-2HG dehydrogenase (*L2HGDH*) genes (Duran et al., 1980; Kranendijk et al., 2012; van der Knaap et al., 1999).

L-2HG has garnered recent interest as elevated levels are observed in several other settings besides inborn errors of metabolism. Multiple studies have demonstrated that L-2HG can be elevated in the setting of hypoxia (Oldham et al., 2015). Additionally, profound increases in L-2HG have been identified in *Drosophila* larval development (Li et al., 2017; Li et al., 2018). L-2HG can be created from α-ketoglutarate (α-KG) by off-target reactions of several enzymes, including lactate dehydrogenase and malate dehydrogenase 1 and 2 (MDH1/2) (Nadtochiy et al., 2016; Intlekofer et al., 2017; Rzem et al., 2007). L2HGDH activity serves to counter this off-target activity and therefore has been referred to as an enzyme of metabolic repair. In addition, L-2HG levels are elevated in the most common histology (clear cell) of renal cell carcinoma (RCC) owing to loss of expression of L2HGDH. Restoration of L2HGDH activity in several RCC models leads to reduced L-2HG levels. Moreover, reducing L-2HG impedes tumor growth *in vivo*, indicating that L-2HG promotes RCC growth. The *L2HGDH* gene is located on chromosome 14q, a region commonly deleted in RCC. As such, RCC tumors that exhibit 14q loss demonstrate reduced L2HGDH expression (Shelar et al., 2018; Shim and Sudarshan, 2015; Shim et al., 2014).

The cellular effects resulting from oncometabolite elevation have been the subject of interest since their initial identification. L-2HG, like other oncometabolites, is structurally similar to α-KG [also referred to as 2-oxoglutarate (2-OG)]. α-KG is a metabolite of the TCA cycle. Additionally, it serves as a co-factor for 2-OG-dependent dioxygenases. These enzymes mediate diverse processes including RNA, DNA and histone demethylation. As such, prior studies have demonstrated increased DNA/RNA/histone methylation in oncometabolite-related tumors with corresponding gene expression changes (Xu et al., 2011; Su et al., 2018; Shim et al., 2014). More recently, D-2HG has been shown to inhibit enzymes that utilize α-KG as a substrate, namely branched-chain aminotransferases (BCAT1/2) that metabolize branched-chain amino acids (McBrayer et al., 2018). These data suggest that oncometabolites can act via effects on gene expression as well as direct effects on metabolism. Furthermore, several factors can impact the effects of an oncometabolite, such as the type of oncometabolite, the α-KG level, the amount of target enzyme and the affinity of the oncometabolite compared with α-KG. Thus, the effect could be tissue specific and can fluctuate based on available nutrients.

Biallelic mutation of *L2HGDH* results in L-2HG aciduria, a neurometabolic inborn error of metabolism marked by neurologic deterioration and decreased life expectancy (Rzem et al., 2007). Notably, it is also associated with the development of brain tumors (Haliloglu et al., 2008). Recent studies have looked at the effects of whole-body *L2hgdh* knockout (KO) mouse models. Rzem et al. (2015) used a gene-trap cassette method that identified that L-2HG inhibits lysine α-ketoglutarate reductase/saccharopine dehydrogenase, leading to depletion of saccharopine and glutamine in the brain. Additionally, Ma et al. (2017) used a piggyback transposon gene insertion method to disrupt *L2hgdh*, and the resulting mice showed extensive brain abnormalities. These studies have provided insight into the role of L2HGDH in the brain. Although L2HGDH is known to prevent L-2HG elevation, the physiologic rationale for keeping L-2HG levels low remains largely unknown. Moreover, as the prior studies focused on brain findings, the teleologic role of this enzyme in other tissues in which it is highly expressed remains unknown. Here, we report the creation of an *L2hgdh* KO model using CRISPR/Cas9 that recapitulates the brain abnormalities noted in prior KO models. Additionally, we demonstrate a role for L2HGDH in mitochondrial metabolism in the kidney, a tissue with high basal L2HGDH expression. These data have implications for the effects of L-2HG in physiologic as well as pathologic states.

## RESULTS

### Generation of L2HGDH KO with CRISPR/Cas9

**Fig. 1.**
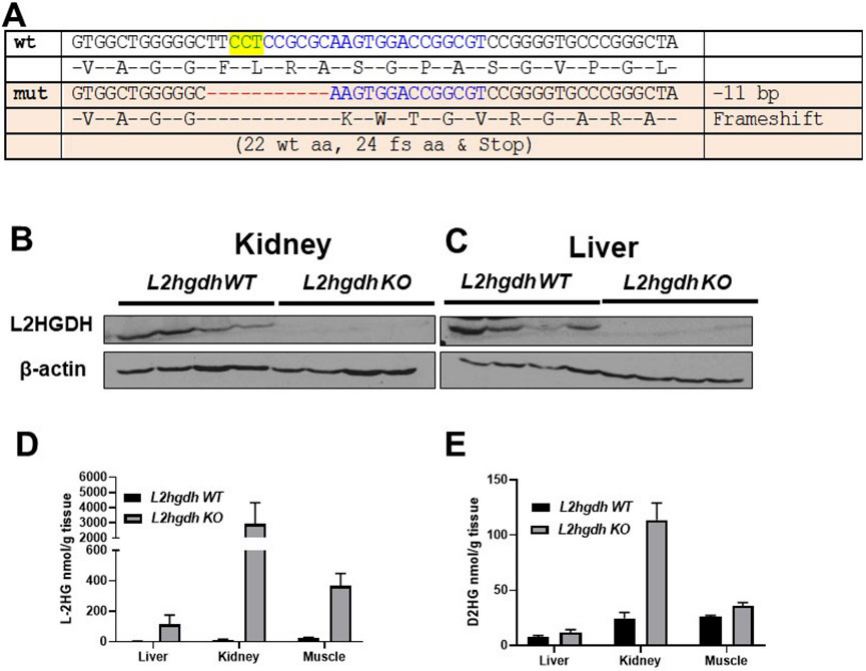
**CRISPR/Cas9** **KO** **of *L2hghd* increases L-2HG levels.** (A) *L2hgdh* sequence of the mouse wild type (wt) and mutant (mut) allele generated, demonstrating 11 bp deletion with resulting frameshift (fs) and premature stop codon. The yellow highlighted region indicates the target protospacer adjacent motif sequence. aa, amino acids. (B,C) Immunoblot for L2HGDH and β-actin in kidney (B) and liver (C) tissue from *L2hgdh* WT and KO mice. (D,E) GC-MS measurements of L-2HG (D) and D-2HG (E) in liver, kidney and muscle tissues. Mice fasted for 12 h prior to initial tissue harvest. Data are means±s.e.m.

CRISPR/Cas9 genome editing was used to generate a deletion in *L2hgdh*, causing a mutant allele with an 11 bp deletion. This deletion results in a frameshift very early in exon 1, encoding the initial 22 amino acids followed by 24 mutant amino acids and a stop codon (Fig. 1A). We inferred this allele to result in complete loss of function of the *L2hgdh* gene. This allele was successfully transmitted through the germline, enabling us to establish a KO line. Protein analysis of both kidney and liver tissues demonstrates loss of *L2hgdh* expression relative to wild-type (WT) animals (Fig. 1B,C). We next analyzed D-2HG and L-2HG levels via gas chromatography–mass spectrometry (GC-MS) in liver, kidney and gastrocnemius (striated) muscle. As heterozygous mice demonstrated comparable L-2HG levels in all three tissues compared with WT (+/+) mice (Fig. S2), littermate control mice included both +/+ and +/− genotypes. Consistent with loss of *L2hgdh* activity, L-2HG levels were elevated in all tissues from KO animals compared with control animals. Moreover, kidney demonstrated a much higher abundance of L-2HG relative to other tissues (Fig. 1D). This result differs from a previous study, which found similar levels of L-2HG among these tissues in KO mice (Ma et al., 2017). Additionally, all three tissues from KO animals demonstrated a modest increase in D-2HG, with kidney tissue demonstrating the highest increase (Fig. 1E).

### Histologic analysis of the brain and kidney

**Fig. 2.**
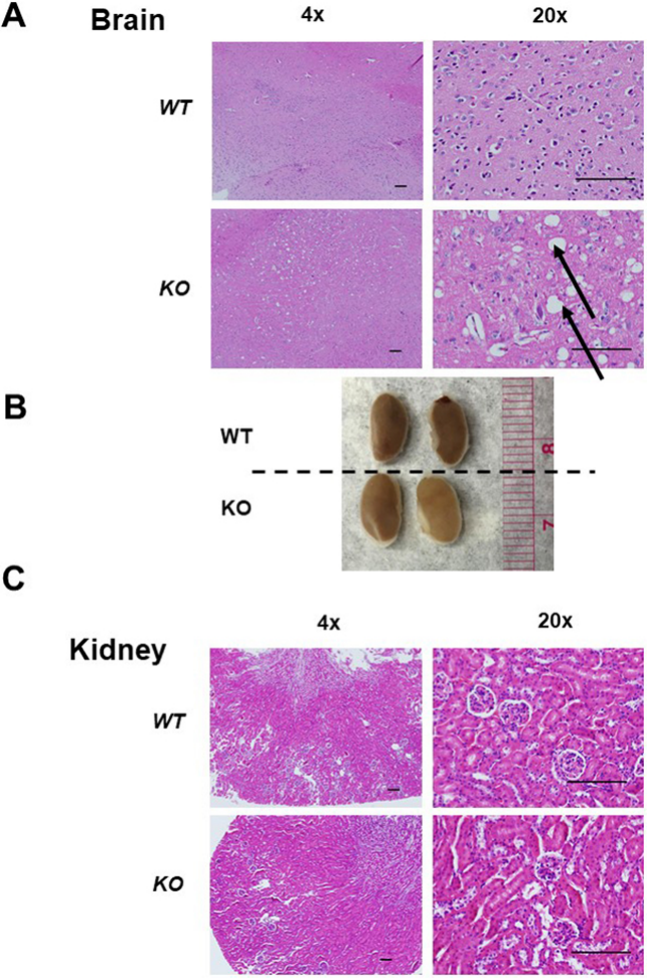
**Histological analysis of *L2hgdh* WT (+/+) and KO (−/−) tissues.** (A) Slices of mouse cerebral cortex stained with H&E. Vacuoles (indicated by black arrows) are noted in *L2hgdh* KO animals. (B) Gross images of kidneys from WT and KO animals. (C) Slices of mouse kidney cortex stained with H&E. Normal-appearing glomeruli and proximal tubules can be identified in both WT and KO mice. Images displayed are at 4× and 20× magnification. Scale bars: 200 μm.

Histologic analysis of the brain was performed in 24-week-old mice. Compared with the *L2hgdh^+/+^* mice, the brains of *L2hgdh^−/−^* mice consistently showed spongiotic appearance and perineuronal vacuoles, predominantly seen in the deep layers of the cerebral cortex, consistent with prior KO models reported (Fig. 2A). The examination of the kidney did not demonstrate any significant differences in gross examination (Fig. 2B). Remarkably, no significant histologic difference was noted when comparing the kidneys from the *L2hgdh^+/+^* and *L2hgdh^−/−^* mice (Fig. 2C).

### Decreased fertility in *L2hgdh*^−/−^ male mice

**Fig. 3.**
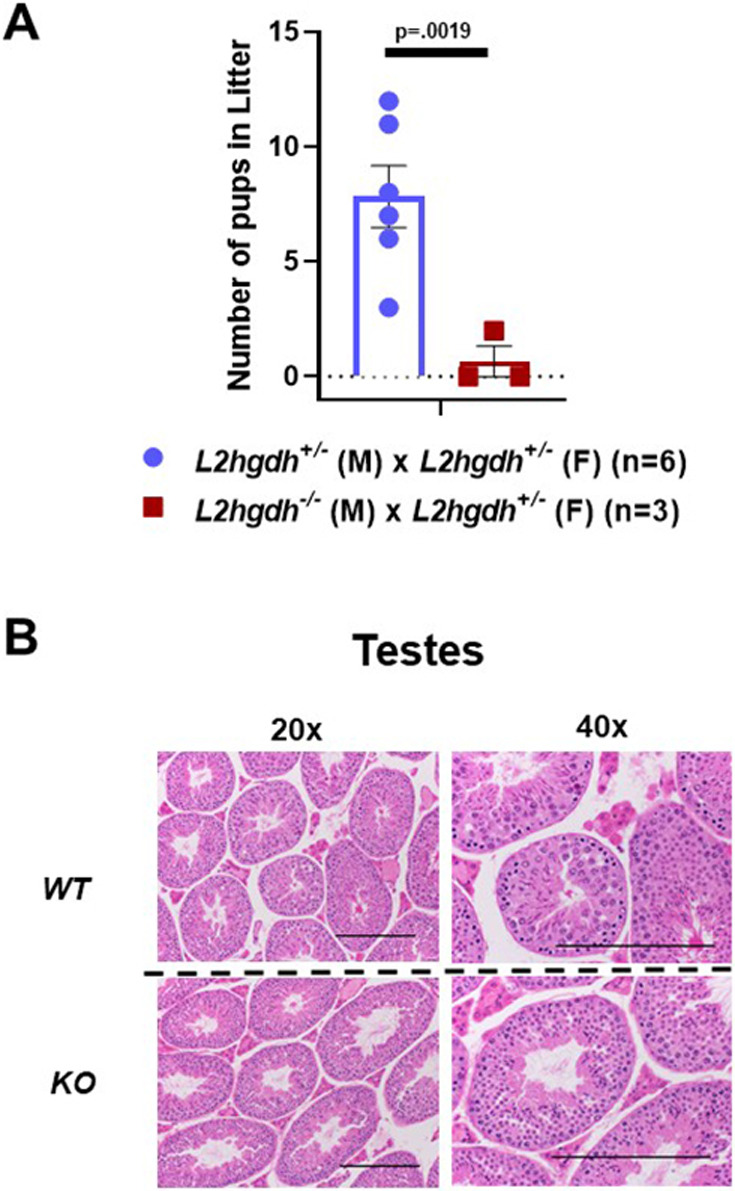
**Fertility analysis of *L2hgdh* heterozygous (+/−) and KO male mice.** (A) Number of pups per litter generated when crossing a heterozygous female (+/−) with either a heterozygous male (+/−) (blue) or homozygous null (−/−) male (red). *n*=number of breeding pairs examined. Graph depicts two-tailed Student's *t*-test results and data are means±s.e.m. (B) Slices of mouse testes after Bouin's fixation and staining with H&E. Images displayed are at 20× and 40× magnification. Scale bars: 200 μm.

We observed that *L2hgdh^−/−^* male mice had significantly reduced fertility when bred with *L2hgdh^+/−^* females (Fig. 3A). Out of three attempts at breeding, only one produced pups. Moreover, this litter had only two pups. This is in stark contrast to the pairing of *L2hgdh^+/−^* mice that produced an average of seven pups per litter. Histologic analysis of testes and accessory sex glands did not demonstrate any differences between WT and KO males (Fig. 3B).

### Reduced succinate in tissues with high L-2HG

**Fig. 4.**
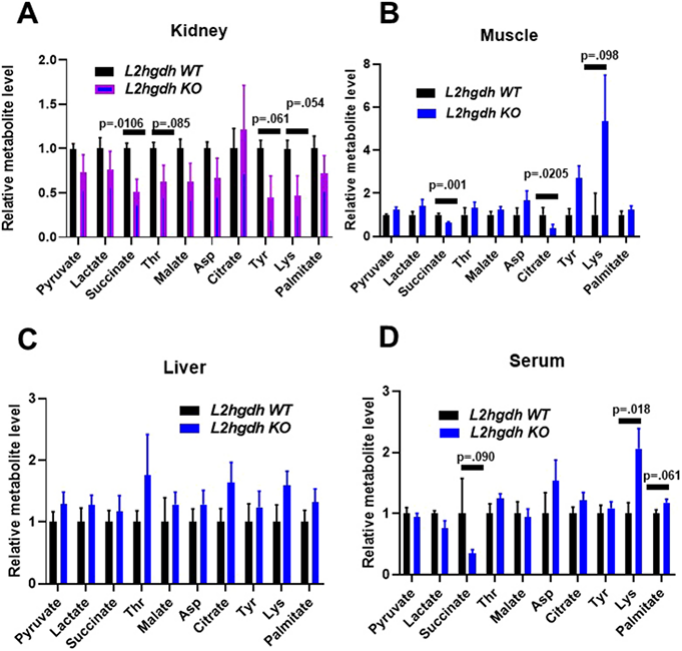
**Metabolite profiling of WT and KO tissues.** GC-MS metabolite profiling of tissues and serum from WT and KO mice. Mice were fasted for 12 h prior to sample harvest and metabolite extraction. Values are normalized to *L2hgdh* WT metabolite amount. *n*=5 mice for each group. Both groups contain both male and female mice. Graphs depict two-tailed unpaired Student's *t*-test results and data are means±s.e.m.

Prior metabolomics studies in *L2hgdh* KO mice focused on the brain (Rzem et al., 2015). However, tissues including kidney, liver and skeletal muscle also express high levels of this enzyme, with the kidney being the highest-expressing tissue in humans (Fig. S2). We therefore performed metabolic profiling in these tissues (in addition to serum) that included small molecules from pathways including glycolysis, the TCA cycle and amino acid metabolism (data provided in Table S1). Notably, TCA cycle metabolites were not measured in previous *L2hgdh* KO mice models. To minimize the potential effects of the diet, we fasted the mice for 12 h before tissue isolation. (*n*=5/group including both male and female mice). Analysis of tissues from KO mice demonstrated significantly decreased succinate in both kidney (*P*=0.0106) and muscle (*P*=0.001). Additionally, this decrease in succinate was trending toward significance in serum as well (*P*=0.090) (Fig. 4). There were also several amino acids (threonine, tyrosine, and lysine) trending toward decreased levels in KO kidneys, for which *P*-values were approaching significance (Fig. 1A).

### L2HGDH restoration promotes TCA cycle flux

**Fig. 5.**
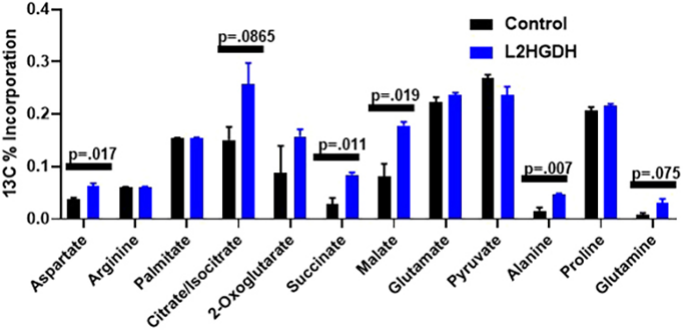
**Metabolite flux analysis of 769-P RCC cells** **with or without** **L2HGDH,** **incubated with [U-^13^C] glucose.** 769-P RCC cells were stably transduced with control vector or L2HGDH. Cells were then treated with [U-^13^C] glucose for 24 h followed by metabolite extraction and LC-MS profiling. Values represent total ^13^C label incorporation into the indicated metabolite. Graph depicts two-tailed unpaired Student's *t*-test results and data are means±s.e.m.

Succinate is a known metabolite of the TCA cycle and is generated by the enzyme succinyl-CoA ligase. As decreased succinate was observed in multiple KO tissues, we considered the possibility of whether elevations of L-2HG could perhaps impact the TCA cycle. From previous studies, we found that 769-P RCC cells had reduced L2HGDH expression with concomitant L-2HG elevation (Shelar et al., 2018). We have previously demonstrated that the restoration of L2HGDH expression significantly lowers L-2HG levels. We therefore assessed the effects of L2HGDH on the TCA cycle. 769-P cells (with or without L2HGDH) were then cultured with fully labeled glucose [U-^13^C] before analysis via liquid chromatography–mass spectrometry (LC-MS). For the analysis, we measured total label incorporation for metabolites of interest. We did not observe a significant difference in label incorporation into citrate/isocitrate or α-KG (Fig. 5). However, we observed that L2HGDH restoration led to increased label incorporation into the TCA cycle metabolites succinate (*P*=0.011) and malate (*P*=0.019) (Fig. 5). We also observed that L2HGDH led to increased label incorporation into amino acids including aspartate (*P*=0.017) and alanine (*P*=0.007). Notably, aspartate is known to be generated by transamination of the TCA cycle intermediate oxaloacetate. No significant difference in glucose (both labeled and unlabeled) was observed as a function of L2HGDH (Table S2).

### Impact of L-2HG on the TCA cycle

As noted, α-KG-dependent dioxygenases are known to catalyze the demethylation of DNA, RNA and histones. Therefore, oncometabolites could impact gene expression via effects on transcription and/or protein translation. Prior analyses demonstrated reduced expression of TCA cycle enzyme expression in RCC specimens (Cancer Genome Atlas Research Network, 2013; Wettersten, 2020). We therefore considered whether L-2HG suppressed the expression of TCA cycle enzymes, thereby leading to reduced TCA cycle flux. We restored L2HGDH (both WT and A241G mutant) in 786O and 769-P RCC cells (Fig. S3). As expected, WT L2HGDH lowered L-2HG, whereas the mutant did not affect L-2HG levels (Fig. S4A,B). In both cell lines, modulation of L-2HG levels had no impact on TCA cycle enzyme mRNA or protein expression (Fig. S4C-E).

**Fig. 6.**
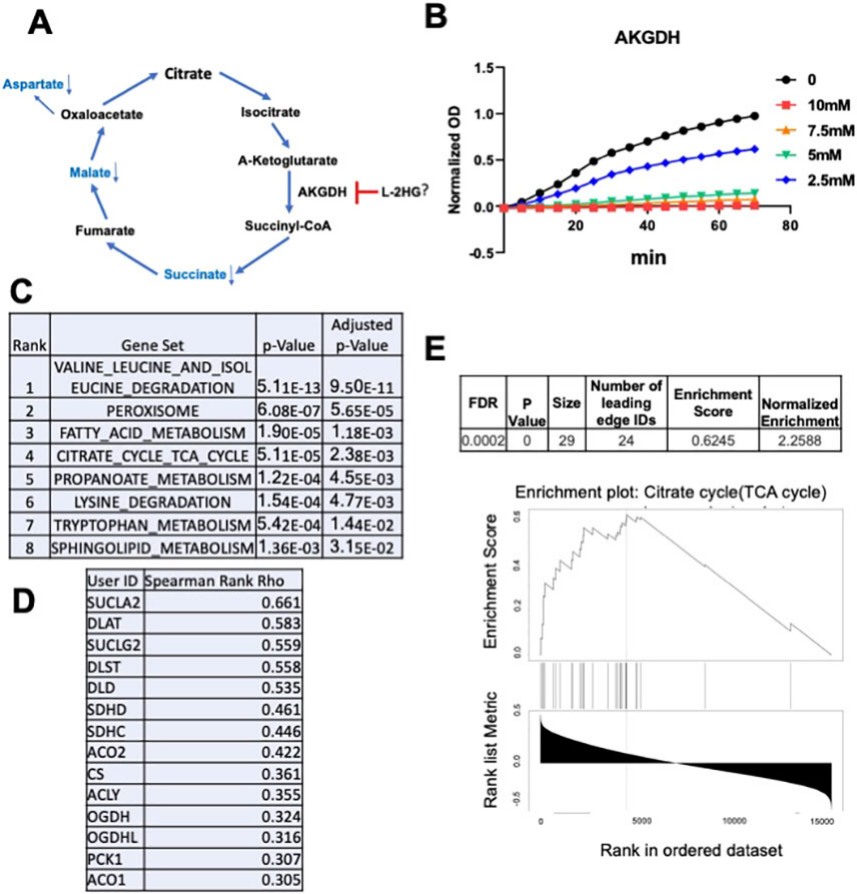
**The L-2HG/L2HGDH axis and the TCA cycle.** (A) Schematic of the proposed effects of L-2HG on the TCA cycle based on flux analysis. (B) Effects of increasing L-2HG levels on α-KGDH enzymatic activity *in vitro*. α-KGDH enzymatic product formation was measured based on optical density (OD) at 450 nm. (C) KEGG (https://www.genome.jp/kegg/) pathway analysis of L2HGDH positively correlated genes in clear cell RCC with Spearman Rank >0.3 (moderate association). Co-expression analysis performed by GRACE (https://grace.biohpc.swmed.edu/). Pathway analysis performed by Enrichr (https://amp.pharm.mssm.edu/Enrichr/). Data are from the TCGA Kidney Renal Clear Cell Carcinoma (KIRC) data set. (D) TCA cycle genes positively correlated with *L2HGDH* from the KEGG TCA cycle pathway in C. (E) Enrichment plot of KEGG TCA cycle using all genes from KIRC *L2HGDH* GRACE analysis. Analysis was performed by Webgestalt (http://www.webgestalt.org/) utilizing Spearman Rank Rho for rank order.

We, therefore, considered alternative mechanisms by which L-2HG could impact the TCA cycle. Recent studies demonstrate that D-2HG can inhibit enzymes that utilize α-KG as a substrate. Within the TCA cycle, α-KG dehydrogenase (α-KGDH) converts α-KG to succinyl-CoA, which is subsequently converted to succinate. In turn, succinate is converted to other TCA cycle intermediates (e.g. malate) as well as metabolites derived from TCA cycle intermediates (e.g. aspartate). Based on our findings related to the TCA cycle, we hypothesized that L-2HG could directly inhibit the enzymatic activity of α-KGDH (Fig. 6A). Using an *in vitro* biochemical assay, we observed that L-2HG led to a dose-dependent inhibition of α-KGDH enzymatic activity (Fig. 6B).

These biochemical data led us to surmise that the teleological role for L2HGDH in the kidney is to keep L-2HG levels low such that TCA cycle flux is maintained. To further support this role for L2HGDH, we examined co-regulated genes within The Cancer Genome Atlas (TCGA) data set on clear cell RCC (KIRC) using the Genomic Regression Analysis of Coordinated Expression (GRACE) tool (Cai et al., 2017) (Table S3). This algorithm performs co-expression analyses while accounting for the potential effects of copy number alterations, which commonly occur in cancers. This is particularly relevant as copy number loss of *L2HGDH* is found in 42.5% of clear cell RCC patients based on TCGA analysis (data not shown). Using a Spearman Rho rank threshold of 0.3 (at least moderate association), we performed a pathway analysis of positivity associated genes with the Enrichr tool (https://amp.pharm.mssm.edu/Enrichr/). This analysis demonstrated that *L2HGDH* is coordinately expressed with genes involved in pathways including branched-chain amino acid degradation, peroxisomes, fatty acid metabolism and the TCA cycle (Fig. 6C). TCA cycle genes that positively correlate with *L2HGDH* are shown in Fig. 6D. Utilizing the Spearman Rank Rho for rank order of genes from the GRACE analysis, we performed pathway analysis utilizing all genes with the Webgestalt tool. Fig. 6E displays the TCA cycle enrichment plot from the analysis. Both the false discovery rate (FDR) and *P*-value were <0.001. These bioinformatics analyses provide further evidence on the role of L2HGDH in promoting TCA cycle metabolism, which corresponds with our biochemical studies.

### An alternative mechanism for reduced L2HGDH and elevated L-2HG

**Fig. 7.**
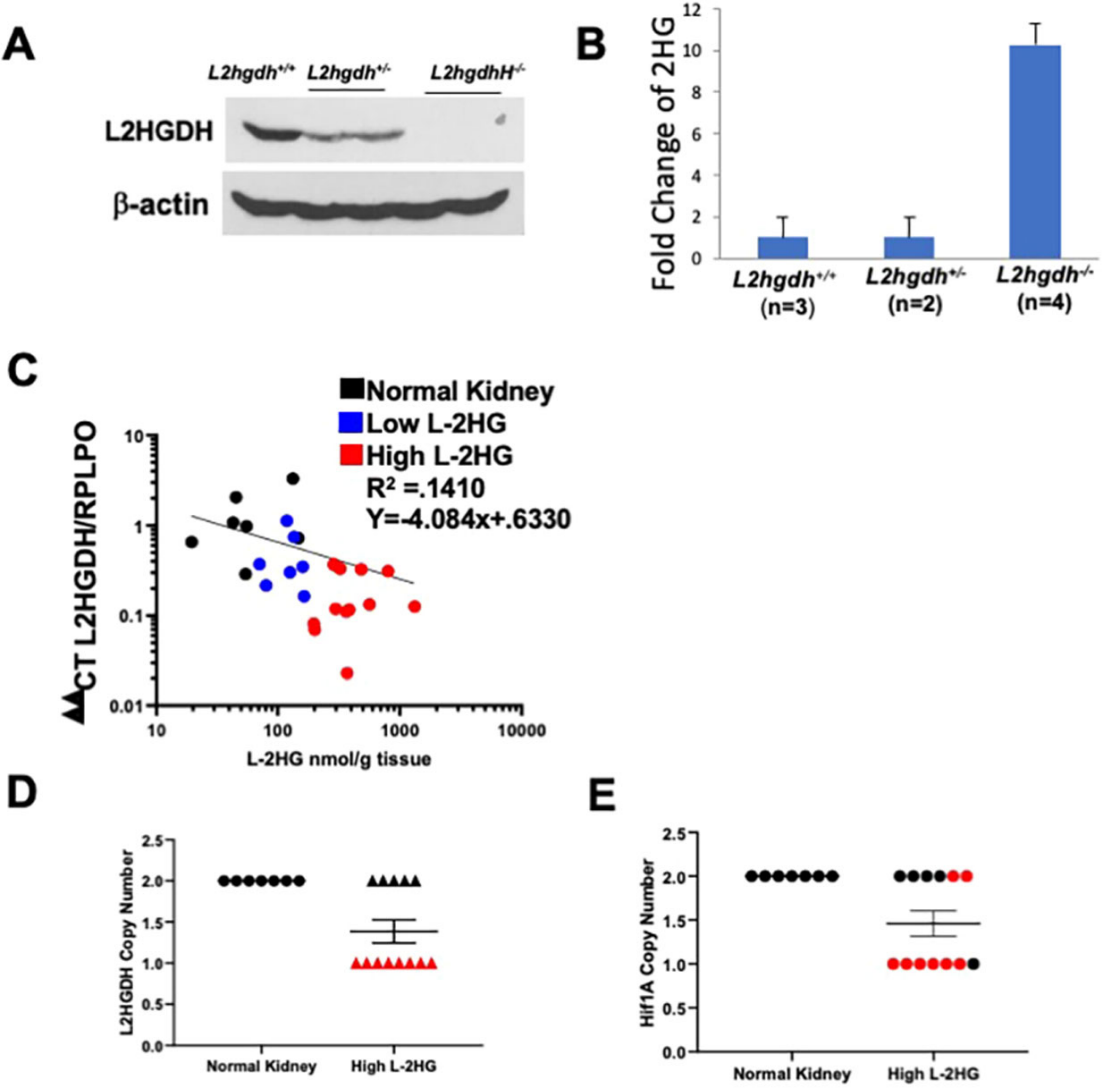
**Alternative mechanisms contribute to decreased *L2HGDH* mRNA in kidney cancer.** (A) Immunoblot of L2HGDH in kidneys from *L2hgdh^+/+^*, *L2hgdh^+/−^* and *L2hgdh^−/−^* mice. (B) Renal 2HG (normalized) from mice with the indicated genotype. (C) *L2HGDH* mRNA (*x*-axis) and L-2HG metabolite levels (*y*-axis) in RCC tumors and normal kidney (black). RCC tumor samples were designated as either low L-2HG (blue) or high L-2HG (red). Low L-2HG designation is based on metabolite level within two standard deviations away from normal kidney median L-2HG levels. Correlation line created in GraphPad Prism 8 using log-log line non-linear fit. (D,E) Copy number analysis by qPCR for *L2HGDH* (D) and *HIF1A* (E) in normal kidney and high L-2HG renal tumors. Both genes are located on 14q. Red values depict tumors with copy loss for *L2HGDH*.

L-2HG build-up in RCC is caused by loss of L2HGDH expression. Our laboratory has previously reported that 14q loss, the chromosomal location for the *L2HGDH* gene, is significantly associated with decreased *L2HGDH* mRNA. Notably, heterozygous loss of *L2hgdh* in mice (*L2hgdh^+/−^*) resulted in reduced L2HGDH protein (Fig. 7A) but was not sufficient to significantly increase 2HG levels in the kidney (Fig. 7B). Only homozygous loss of *L2hgdh* led to increased 2HG in the kidney. These data suggest that although gene copy loss can reduce L2HGDH expression, this mechanism alone does not result in raised L-2HG, and that alternative mechanisms exist that reduce L2HGDH in RCC to the point that L-2HG becomes elevated.

As further supportive evidence, we analyzed *L2HGDH* mRNA expression and gene copy number in normal kidney and RCC specimens. RCC specimens were separated into high and low L-2HG tumors based on whether levels were within two standard deviations of normal kidney median. A log-log plot of linear regression analysis identified a negative correlation with a R^2^ of 0.1410 (Fig. 7C). Next, we analyzed high L-2HG tumors for *L2HGDH* copy loss. Although eight of 13 high-L-2HG tumors demonstrated *L2HGDH* copy loss, we noted five tumors with raised L-2HG without evidence of copy loss (Fig. 7D). As a control for our copy number analysis, we examined *HIF1A*, which is also located on 14q. Of the eight high-L-2HG tumors that demonstrated *L2HGDH* copy loss (monoallelic loss), six tumors also demonstrated *HIF1A* copy loss (Fig. 7E). Correspondingly, of the five high-L-2HG tumors without *L2HGDH* copy loss, four tumors did not demonstrate *HIF1A* loss. Collectively, these data further support our findings that alternative mechanisms can promote loss of L2HGDH expression with ensuing elevated L-2HG in RCC.

**Fig. 8.**
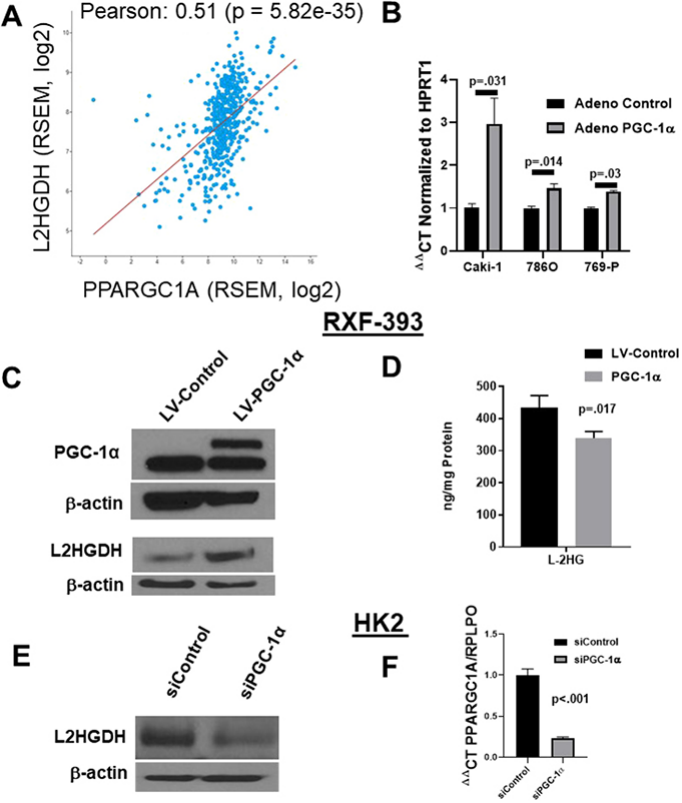
**PGC-1α regulates L2HGDH expression.** (A) TCGA KIRC (clear cell RCC) correlation plot comparing RNA-Seq by Expectation Maximization (RSEM) RNA values for *PPARGC1A* and *L2HGDH* using cBIO analysis portal (http://www.cbioportal.org/). (B) *L2HGDH* mRNA expression was assessed in RCC lines following adenoviral delivery of PGC-1α relative to control adenovirus. (C) Immunoblot analysis for L2HGDH following stable lentiviral (LV) expression of PGC-1α in RXF-393 RCC cells. In the PGC-1α immunoblot, the lower band represents the non-specific band. (D) L-2HG levels in RXF-393 cells with or without PGC-1α. (E) Immunoblot for L2HGDH in HK2 renal epithelial cells following transfection with the indicated siRNA. (E) RT-qPCR for *PPARGC1A* following treatment with the indicated siRNA in HK2 cells. Graphs depict two-tailed unpaired Student's *t*-test results and data are means±s.e.m.

Based on the combined analysis of our KO model and biospecimens, we sought to identify alternative mechanisms for reduced L2HGDH/elevated L-2HG in RCC. Utilizing our information from pathway analysis of genes co-regulated with *L2HGDH*, we attempted to identify potential transcription factors that are known to regulate mitochondrial metabolic pathways and that are reduced in RCC. One such transcription factor identified was the gene *PPARGC1A*, which encodes peroxisome proliferator-activated receptor gamma coactivator 1-alpha (PGC-1α). Our laboratory recently reported on reduced PGC-1α in RCC tissues and multiple RCC lines with reduced expression (Nam et al., 2020). Analysis of TCGA data on clear cell RCC demonstrates that *PPARGC1A* and *L2HGDH* mRNA are positively correlated (Fig. 8A). Restoration of PGC-1α expression via adenovirus increased the mRNA expression of *L2HGDH* in multiple RCC lines examined (Fig. 8B). Furthermore, stable expression of PGC-1α via lentiviral transduction in RXF-393 cells increased L2HGDH protein and significantly reduced L-2HG levels (Fig. 8C-D). Additionally, knockdown of *PPARGC1A* utilizing small interfering RNA (siRNA) decreased L2HGDH protein in both HK2 renal epithelial cells and HEK293T embryonic kidney cells (Fig. 8E; Fig. S5). *PPARGC1A* knockdown was confirmed by quantitative PCR (qPCR) (Fig. 8F). Collectively, these data demonstrate a role for PGC-1α in regulating L2HGDH expression and L-2HG levels.

## DISCUSSION

L-2HG can have widespread effects on a cell. L-2HG has been shown to competitively inhibit enzymes that utilize α-KG as a co-factor with effects on DNA and histone methylation. Finding the models to reflect the most impactful effects of L-2HG accurately is essential to better understand its role in cancer, hypoxia and development. There is an abundance of different techniques that can be used for *in vivo* gene-edited mouse models. Recent studies have shifted to CRISPR/Cas9 for both its ease to generate models and its specificity (Weber and Rad, 2019). We thus compared our new model to previous models and found similar characteristics. All models showed increased L-2HG in all tissues tested and abnormal pathology in brain tissue (Rzem et al., 2015; Ma et al., 2017). Unlike previous models, our *L2hgdh* KO mouse model demonstrated more pronounced L-2HG elevation in the kidney compared with the liver and muscle. Moreover, KO males showed significantly reduced fertility (Fig. 3). Testes are also known to express L2HGDH. Notably, rodent testes express a variant of LDH-C, which can produce high levels of L-2HG (Teng et al., 2016).

We used this model to examine the biochemical implications of raised L-2HG in the kidney, the tissue with the highest expression of L2HGDH in humans (Fig. S2). We used a metabolomic approach to study the effects of L-2HG. Our GC-MS analysis of the kidney and muscle tissues demonstrated a significant decrease in succinate levels compared with control mice (*P*=0.01 and *P*=0.001, respectively), leading us to hypothesize a link between L-2HG and the TCA cycle. Interestingly, KO liver did not show a significant difference in succinate levels. One possible explanation is that the degree of L-2HG elevation in KO liver was less than that observed in kidney and muscle (Fig. 1D). Alternatively, these data could indicate that the effects of L-2HG are tissue dependent.

The link between L-2HG and the TCA cycle is particularly intriguing as RCC tumors are known to demonstrate reduced expression of TCA cycle enzymes (Cancer Genome Atlas Research Network, 2013). Correspondingly, isotope labeling studies with [U-^13^C] glucose in patients (Courtney et al., 2018) provided a reference for interpretation of our analyses. The authors demonstrate reduced TCA cycle labeling in RCC relative to normal kidney. Reintroduction of L2HGDH to RCC cells led to increased ^13^C labeling of TCA cycle metabolites, including succinate and malate, as well as metabolites derived from the TCA cycle such as aspartate. Interestingly, there were no significant differences in the labeling of TCA metabolites earlier in the cycle (citrate/isocitrate and α-KG). Similarly, reduced labeling in TCA cycle metabolites succinate, fumarate and malate, but not in metabolites earlier in the cycle, such as citrate, was observed (Courtney et al., 2018). These data led us to focus on α-KGDH. As further evidence for a link between L-2HG and α-KGDH, bacterial species of *Marinomonas* often demonstrate the clustering of αKGDH subunit genes and *ygaF*, the gene encoding the bacterial equivalent of L2HGDH (Seaver et al., 2012). These data reinforce the role of L2HGDH as an enzyme of metabolite repair by keeping L-2HG levels ‘in check’, such that α-KGDH activity is maintained. One caveat to our studies is that alternative carbon sources can contribute to TCA cycle anaplerosis. This is particularly relevant as glucose entry into the TCA cycle is impaired in RCC due to HIF1A-mediated inhibition of pyruvate dehydrogenase (Papandreou et al., 2006; Kim et al., 2006). For example, glutamine can be metabolized to α-KG. Hence, raised L-2HG could impact the anaplerotic role of these alternative carbon sources.

Our data demonstrate that L-2HG promotes TCA cycle dysfunction and provide new insight into the mechanisms that lead to a classic Warburg phenotype in RCC. Despite these data, the true biological significance of L2HGDH remains poorly characterized. Nevertheless, they may provide new opportunities for intervention. The TCA cycle represents a major bioenergetic hub with cataplerotic activities that are known to have biosynthetic functions. Hence, raised L-2HG may create potential metabolic liabilities that could be used for treating tumors. Alternatively, potential deficiencies resulting from these metabolic liabilities could be treated with supplementation in patients with 2HG acidurias.

Our study has provided new insight into the regulation of L2HGDH based on our finding that biallelic loss of *L2hgdh* was required to raise renal L-2HG levels. These data indicate that single-copy loss of *L2HGDH* alone is not sufficient to lower L2HGDH expression to the point that L-2HG levels are increased in kidney tumors. As further evidence, we identified several renal tumors with low *L2HGDH* expression/elevated L-2HG without *L2HGDH* copy loss. These data allude to the importance of L-2HG in RCC in that reduced *L2HGDH* expression is not merely a bystander effect associated with copy loss, but instead suggest that there is a concerted effort to silence this gene in RCC.

Our results also provide new insight into the role of PGC-1α in mitochondrial metabolism. PGC-1α has been shown to promote oxidative metabolism via transcriptional regulation of genes encoding oxidative phosphorylation subunits and TCA cycle enzymes (LaGory et al., 2015; Calvo et al., 2008; Koves et al., 2005; Bhalla et al., 2011). Our data demonstrate that PGC-1α also promotes TCA cycle metabolism via *L2HGDH* transcription, which lowers L-2HG levels. One caveat of our experiments is that all cell lines examined have a heterozygous loss of *L2HGDH* (Fig. S1). As a result, the effects of PGC-1α on *L2HGDH* transcription might be dampened. Although L-2HG does not have effects on the expression of TCA cycle enzymes in RCC, it can affect TCA cycle metabolism. As a result of low L-2HG levels, α-KGDH activity is maintained, thereby promoting TCA cycle flux. These data, therefore, indicate that the teleological role for L2HGDH, at least in the kidney, is to promote mitochondrial metabolism, which is in line with our biochemical findings. Our findings are consistent with previous work demonstrating that high D-2HG levels can lower α-KGDH activity in cardiac muscle tissue. However, the direct inhibitory role of D-2HG on α-KGDH enzymatic activity was not examined (Karlstaedt et al., 2016). These data are in line with prior studies that have demonstrated that both enantiomers of 2HG can inhibit enzymes that utilize α-KG as a co-factor or substrate. Our study focused on L-2HG because D-2HG is not significantly elevated in RCC. Further studies on the effects of D-2HG on TCA cycle metabolism in IDH mutant tumors would be of interest.

In summary, this paper highlights the creation and analysis of a novel *L2hgdh* KO mouse model in concert with human RCC tissue analysis to study the metabolite L-2HG. We demonstrate a role for L2HGDH in promoting mitochondrial metabolism in the kidney. These data provide new insights into the pathophysiological implications of raised L-2HG and could provide new avenues for therapeutic intervention.

## MATERIALS AND METHODS

### Lentivirus

L2HGDH (WT) and L2HGDH mutant (A241G) complementary DNA (cDNA) have been previously described (Shelar et al., 2018). To generate stable cell lines, lentiviral plasmids were transfected with packaging vectors into HEK293T cells using the calcium chloride method. Supernatants from transfected HEK293T cells were collected after 72 h, filtered and applied to cells. Viral transduced cells were selected in culture medium containing puromycin. All transduced cells represent polyclonal populations.

### Adenovirus

*PPARGC1A* (encoding PGC-1α) and GFP control adenovirus were purchased from Vector Biolabs. Cells were analyzed 48-72 h after transduction. A multiplicity of infection (MOI) of 100 was used for all experiments.

### siRNA studies

Cells were seeded on six-well plates for 48 h. Cells were then transfected with 50 nM of a negative control siRNA or siRNA against PGC-1α using Lipofectamine^®^ RNAiMAX regent (Invitrogen) for 48 h. Additional methods were as described previously (Nam et al., 2020).

### Generation of L2HGDH KO mice

In order to create loss-of-function alleles of the mouse *L2hgdh* gene (ENSMUSG00000020988), CRISPR targets were chosen in the coding region of exon 1 (ENSMUSE00000113742; Transcript ID ENSMUST00000021370.9) using the Massachusetts Institute of Technology CRISPR design tool (https://zlab.bio/guide-design-resources). Two guide sequences with high scores that indicated a low number of off-target sites were chosen: C1, AGACACCGCCTACGTAGCGC(AGG); and C2, ACGCCGGTCCACTTGCGCGG(AGG). Guide RNA molecules were generated using the method described by Hwang et al. (2013). Cas9 mRNA was prepared by *in vitro* transcription using a linearized pCS2-nCas9n plasmid template (Jao et al., 2013).

### Genotyping G0/founders and F1 animals to identify mutant alleles

Genotyping by PCR-heteroduplex mobility assay (HMA) was employed to identify indels in the G0/founder animals (Challa et al., 2016), using the following primers: forward 5′-CCTTAGAGTCCGTTCAGGTTG-3′; reverse 5′-GGACACAGACAGGTTCAGTTG-3′, which amplified a 271 bp fragment. The PCR amplicons were cloned and sequenced using the Sanger method to obtain the mutant allele information.

### Pathology

Reproductive tissues were fixed in Bouin's solution before being processed. All other tissues were formalin fixed before processing by the University of Alabama at Birmingham (UAB) Comparative Pathology Laboratory. Fixed tissue was placed onto slides and stained with Hematoxylin and Eosin (H&E) for analysis.

### Mouse tissue GC-MS

Mice were fasted for 12 h before tissue isolation. Tissues were briefly washed in ice-cold Dulbecco's PBS, followed by rapid freezing in liquid nitrogen. Metabolite extraction and derivatization were conducted using a modified version of previously described protocols (Chan et al., 2011; Gibson et al., 1993; Pasikanti et al., 2008). Briefly, samples were added to pre-tared 2 ml screw cap tubes containing 1.4 mm ceramic beads, massed, and 800 µl of −20°C methanol with 2 µg/ml of both d4-succinic acid and disodium (R,S)-[2,3,3-2H3]-2-hydroxyglutarate ([2H3]-2HG) (C/D/N Isotopes, Canada) was added to the tubes. Samples were homogenized in an Omni Beadruptor 24 for 30 s at 6.45 m/s, returned to a −20°C benchtop enzyme cooler, and incubated at −20°C for 1 h. Samples were then centrifuged at ∼20,000 ***g*** in a refrigerated centrifuge for 5 min to pellet insoluble debris. The supernatant was removed, evenly split between two tubes and dried in a vacuum centrifuge. One tube was derivatized using a previously described method to quantify D-2HG and L-2HG levels. The second tube was derivatized using methoxylamine hydrochloride (MOX) and N-methyl-N-trimethylsilyltrifluoracetamide (MSTFA) to measure the relative abundance of organic acids, amino acids and glycolytic intermediates. Derivatized samples were injected into an Agilent 7890B/7250 GC-QTOF instrument (1:10 split ratio) equipped with a Phenomex ZB5-5 MSi column using a Gerstel MPS autosampler using previously described methods (Li and Tennessen, 2018). Data were analyzed using MassHunter Qualitative Analysis and MassHunter Quantitative Analysis. Power analysis based on preliminary data was calculated with mu1: 1, mu2: 1.5, sigma of 0.25 alpha: 0.05, and power: 0.8, demonstrating a minimal need for four mice. We used *n*=5 mice in the analysis. Both groups included both male and female mice.

### Cell culture

Renal cell lines were acquired from American Type Culture Collection (ATCC) except for RXF-393 [obtained from the National Cancer Institute (NCI)]. Cells acquired from ATCC and the NCI were characterized via short tandem repeat (STR) profiling. As cells were passaged for less than 3 months after resuscitation and were periodically screened for *Mycoplasma* using a PCR-based assay, no further authentication was performed. HEK293T and Caki-1 cells were maintained in Dulbecco's modified Eagle medium containing 10% fetal bovine serum (FBS) and penicillin/streptomycin. RXF-393, 769-P and 786O cells were maintained in RPMI containing 10% FBS and penicillin/streptomycin. Phenotype/genotype information for cell lines used including L2HGDH expression, *VHL* status and copy number status is provided in Fig. S1.

### Bioinformatics/TCGA analysis

Co-expression analysis of TCGA data on clear cell renal cancer (KIRC) was performed using the GRACE analysis tool (https://grace.biohpc.swmed.edu/) (Cai et al., 2017) as well as the cBIO analysis portal (www.cbioportal.org) (Gao et al., 2013; Cerami et al., 2012). Kyoto Encyclopedia of Genes and Genomes (KEGG) (https://www.genome.jp/kegg/) (Kanehisa and Goto, 2000; Kanehisa et al., 2019; Kanehisa, 2019) was used for pathway mapping. Pathway analysis was performed by Enrichr (Chen et al., 2013; Kuleshov et al., 2016) (https://amp.pharm.mssm.edu/Enrichr/). Enrichment analysis was performed by Webgestalt (http://www.webgestalt.org/) (Liao et al., 2019; Wang et al., 2017; Wang et al., 2013; Zhang et al., 2005).

### α-KGDH assay

The enzymatic assay was obtained from BioVision. For cell-independent experiments, α-KGDH enzyme and necessary co-enzymes were incubated with increasing concentrations of L-2HG. L-2HG was synthesized as previously described (Shim et al., 2014). Optical density readings at 450 nm were taken every 5 min over 70 min.

### Tumor copy number analysis

Clear cell RCCs and normal tissues were acquired from the Cooperative Human Tissue Network. DNA was isolated from tissues using a Qiagen DNeasy Blood and Tissue Kit. Gene copy number was determined using commercially available TaqMan Copy Number assays with fluorescein amidite dye-labeled probes: Hs02530250_cn (*HIF1A*), and Hs07069935_cn (*L2HGDH*) (Thermo Fisher Scientific). The TERT TaqMan copy number reference with a VIC dye-labeled probe (4403316) was used as the two-copy reference (Thermo Fisher Scientific). PCR reactions were performed in triplicate in a 20 μl reaction containing 80 ng DNA, 1× TaqMan Universal PCR Master Mix (Thermo Fisher Scientific), 1× gene-specific Copy Number Assay and 1× Copy Number Reference Assay. The reactions were cycled at 95°C for 10 min, followed by 40 cycles of 95°C for 15 s and 60°C for 1 min in a Bio-Rad CFX96 Touch Real-Time PCR system. Cq (Ct) values were determined using the CFX Manager software (Bio-Rad). Gene copy numbers for the test samples were determined using the 2^−ΔΔCq^ method with a two-copy calibrator.

### RNA and protein analysis

Total RNA from cultured cells was extracted using Trizol reagent (Invitrogen). cDNA was synthesized using a High-Capacity cDNA Reverse Transcription Kit (Thermo Fisher Scientific). Real-time qPCR was performed using the following TaqMan gene expression assays (Thermo Fisher Scientific): *L2HGDH* (Hs00227575), *PPARGC1A* (Hs00173304_m1), *MDH1* (Hs00936497_g1), *MDH2* (Hs00938918_m1), *ACO2* (Hs00426616_g1) and *OGDH* (Hs01081865_m1). *HPRT1* (Hs02800695_m1) and *RPLPO* (hs99999902_m1) probes were used as an internal control, and the ΔΔCt method was used to calculate relative mRNA levels. For immunoblotting, anti-α-L2HGDH (GeneTex, GTX32695, 1:2000; and Novus, NBP2-85197, 1:1000), anti-PGC-1α (Abcam, ab54481, 1:1000) anti-α-MDH2 (Abcam, ab96193, 1:3000), anti-MDH1 (Novus, NBP1-895151, 1:3000) and anti-β-actin (Abcam, ab20272, 1:3000) were used as per the manufacturers' instructions.

### 2HG enantiomer analysis (i.e. D-2HG and L-2HG quantification)

Samples were analyzed as previously described (Rakheja et al., 2011). Enantiomer analysis was performed following derivatization with diacetyl-L-tartaric acid followed by liquid chromatography–tandem mass spectrometry (LC-MS/MS) analysis and normalized to protein levels.

### Total 2HG measurements

Total 2HG (D-2HG+L-2HG) measurement of samples from renal tissues was performed as previously described (Shim et al., 2014). Briefly, tissues were washed in PBS, followed by extractions with 10% cold trichloroacetic acid (TCA). Following centrifugation and removal of the precipitate, TCA in the supernatant was removed by vortexing with four volumes of 1,1,2-trichlorotrifluoroethane-trioctylamine (Sigma-Aldrich) mixture. The aqueous layer was collected and analyzed by ion chromatography coupled with negative electrospray mass spectrometry (Dionex).

### LC-MS isotope labeling analysis

Cells were plated with RPMI with 10% dialyzed FBS, and penicillin/streptomycin. After 24 h, [U-^13^C]-glucose was added to the medium for 24 h. Cells were then washed with ice-cold 0.9% NaCl in molecular-grade water. Cells were lysed in 80% LC-MS methanol and scraped into 1.5 cm^3^ conical vials. Cells were then placed in the freezer at −80°C and further processed on dry ice. Cells were spun at full speed for 20 min at 4°C. The supernatant was split between two samples and dry vacuumed at room temperature until no liquid remained. The dry pellets were reconstituted into 30 ml sample solvent (water:methanol:acetonitrile, 2:1:1, v/v) and 3 ml was further analyzed by LC-MS. The liquid chromatography method was as described previously (Liu et al., 2015, 2014), except that mobile phase A was replaced with water containing 5 mM ammonium acetate (pH 6.8). The Q Exactive Plus mass spectrometer is equipped with a heated electrospray ionization probe with related parameters set as below: heater temperature, 120°C; sheath gas, 30; auxiliary gas, 10; sweep gas, 3; spray voltage, 3.0 kV for the positive mode and 2.5 kV for the negative mode; capillary temperature, 320°C; S-lens, 55; scan range (m/z), 70-900 for positive mode (1.31-12.5 min) and negative mode (1.31-6.6 min) and 100-1000 for negative mode (6.61-12.5 min); resolution: 70,000; automated gain control, 3×106 ions. Customized mass calibration was performed before data acquisition. LC-MS peak extraction and integration were performed using commercially available software Sieve 2.2 (Thermo Fisher Scientific). The peak area was used to represent the relative abundance of each metabolite in different samples. The missing values were handled as described previously (Liu et al., 2014).

### Statistical analysis

Statistical analyses were carried out using GraphPad Prism 6 software. Comparisons between groups for statistical significance were performed with two-tailed unpaired Student's *t*-tests with *P*<0.05 considered significant unless otherwise specified.

### Study approval

All mouse experiments were performed following the Guide for the Care and Use of Laboratory Animals published by the National Institutes of Health, and experimental protocols were approved and conducted according to the UAB Institutional Animal Care and Use Committee.
